# Comparing anticipatory and stop-signal response inhibition with a novel, open-source selective stopping toolbox

**DOI:** 10.1007/s00221-022-06539-9

**Published:** 2023-01-13

**Authors:** Corey G. Wadsley, John Cirillo, Arne Nieuwenhuys, Winston D. Byblow

**Affiliations:** 1 Movement Neuroscience Laboratory, Department of Exercise Sciences, The University of Auckland, Auckland, 1023 New Zealand; 2 Centre for Brain Research, The University of Auckland, Auckland, 1023 New Zealand

**Keywords:** Selective stopping, Response inhibition, Cognitive control, Motor control, Open-source

## Abstract

Response inhibition is essential for terminating inappropriate actions and, in some cases, may be required selectively. Selective stopping can be investigated with multicomponent anticipatory or stop-signal response inhibition paradigms. Here we provide a freely available open-source Selective Stopping Toolbox (SeleST) to investigate selective stopping using either anticipatory or stop-signal task variants. This study aimed to evaluate selective stopping between the anticipatory and stop-signal variants using SeleST and provide guidance to researchers for future use. Forty healthy human participants performed bimanual anticipatory response inhibition and stop-signal tasks in SeleST. Responses were more variable and slowed to a greater extent during the stop-signal than in the anticipatory paradigm. However, the stop-signal paradigm better conformed to the assumption of the independent race model of response inhibition. The expected response delay during selective stop trials was present in both variants. These findings indicate that selective stopping can successfully be investigated with either anticipatory or stop-signal paradigms in SeleST. We propose that the anticipatory paradigm should be used when strict control of response times is desired, while the stop-signal paradigm should be used when it is desired to estimate stop-signal reaction time with the independent race model. Importantly, the dual functionality of SeleST allows researchers flexibility in paradigm selection when investigating selective stopping.

## Introduction

Humans rely on response inhibition to stop inappropriate pre-planned or ongoing actions. Response inhibition can be required in nonselective or selective stopping contexts. Nonselective stopping refers to scenarios where all components of a response must be terminated, for example, actions that require coordination of effectors with a common goal. Selective stopping represents complex scenarios where inhibitory control is required in response to only certain stimuli (stimulus-selective; Bissett and Logan 2014) or only part of a multicomponent action (response-selective; Coxon et al. 2007). Selective stopping may better capture the complexity of human actions, which often require the coordination of multiple effectors guided by various environmental stimuli (for a detailed review of selective stopping, see Wadsley et al. 2022b). To date, investigations of selective stopping have provided new insights into healthy ageing (Albert et al. 2022; Coxon et al. 2016; Hsieh and Lin 2017) and may lead to a better understanding of clinical conditions associated with poor impulse control (MacDonald et al. 2016; Rincon-Perez et al. 2020).

Multicomponent stop-signal task (SST; Lappin and Eriksen 1966; Verbruggen et al. 2019) and anticipatory response inhibition (ARI; He et al. 2021; Slater-Hammel 1960) paradigms can be used to investigate nonselective and response-selective stopping (hereby referred to as selective stopping for simplicity). Both paradigms require a default multi-effector action (e.g., press two buttons) in response to a go-signal which, on a subset of trials, must be terminated on the presentation of a stop signal. Responses in the SST are equivalent to a reaction time task where an imperative go-signal cues a speeded action. In contrast, responses in the ARI are cued by a predictable indicator reaching a stationary target. Both paradigms can assess nonselective stopping by presenting a stop-signal to all subcomponents of the cued response (stop-all trials), or they can assess selective stopping by presenting a stop signal to only one subcomponent while the other continues as initially cued by the go-signal (partial-stop trials). Importantly, both a go- and a stop-signal is presented during stop trials, which better captures response inhibition as a form of top-down cognitive control than Go/No-Go paradigms (Verbruggen and Logan 2008).

Selective stopping can be supported by selective or nonselective response inhibition. During partial-stop trials, a substantial response delay is observed in the subcomponent cued to go when the subcomponent cued to stop is successfully withheld (Coxon et al. 2007). This *stopping-interference effect* has been observed across various within-limb and between-limb effector pairings across SST and ARI paradigms (Aron and Verbruggen 2008; MacDonald et al. 2012; Xu et al. 2015). Stopping-interference likely arises due to a restart process during partial-stop trials (De Jong et al. 1995), whereby a response delay reflects nonselective response inhibition followed by selective re-initiation of the effector cued to respond (MacDonald et al. 2017). Importantly, the stopping-interference effect is not hard-wired and can be modulated by factors that result in nonselective response inhibition (e.g., functional coupling; Wadsley et al. 2022a), or vice-versa, factors that allow for selective response inhibition (e.g., proactive cueing; Majid et al. 2012). Therefore, the stopping-interference effect provides a means to capture the selectivity, or lack thereof, of response inhibition.

An independent race model can describe behaviour in response inhibition paradigms (Logan and Cowan 1984). The independent race model proposes a theoretical race between independent go and stop processes triggered by their respective signals. The outcome on a given stop trial is governed by which of the two processes ‘wins’ the race. The covert latency of response inhibition, termed *stop-signal reaction time* (SSRT), can be estimated since both a go- and a stop-signal is presented during a stop trial (Logan et al. 1984). Estimates of SSRT provide an objective measure of stopping latency that can be compared across experimental conditions and populations (e.g., Aron et al. 2003). Importantly, behavioural data needs to meet the independence assumption of the race model (i.e., response times during failed stop trials should be faster than go trials) for SSRT estimates to be reliable (Verbruggen et al. 2019).

The aim of the current study is to improve the ability to conduct research on selective stopping. At present, selective stopping research has been limited by software availability. Open-source versions of the ARI (He et al. 2021) and SST (Verbruggen et al. 2008) paradigms are freely available, however, they do not provide the functionality to assess selective stopping. Current open-source paradigms also only support using either the SST or ARI paradigm, but not both. We believe that the best practice for selective stopping research is to have the option to choose either paradigm and to have maximum flexibility with task design. Therefore, to achieve our aim we have developed a freely available, open-source toolbox to design selective stopping tasks with in-built optionality for either the SST or ARI paradigm. The toolbox was then used to conduct an experiment to provide key comparisons of behavioural data acquired using the SST and ARI paradigms. In doing so, the experiment reveals important information about each paradigm’s strengths and limitations, guiding recommendations for future use and interpretation of findings. Three hypotheses were tested: (1) Response slowing and variation would be greater during the SST than ARI paradigm. (2) The stopping-interference effect would be larger during the SST than ARI paradigm. (3) The assumption of the independent race model would be met more frequently with the SST than the ARI paradigm.

## Methods

### Participants

Participants were eligible for the experiment if they had no history of neurological illness and were aged between 18 and 60 years. Forty neurologically healthy adults volunteered to participate and met the inclusion criteria (23 female and 17 male; mean age 29.4 yrs., range 18 to 56 yrs.; 36 right-handed and 4 left-handed). The study was approved by the University of Auckland Human Participants Ethics Committee (Ref. UAHPEC22709).

### The selective stopping toolbox

The Selective Stopping Toolbox (SeleST) allows response inhibition to be assessed in nonselective and selective stopping contexts via the SST or ARI paradigm. SeleST was developed using freely available Python-based PsychoPy software that provides high-quality stimulus and response timing (Bridges et al. 2020; Peirce et al. 2019). SeleST is open-source and made freely available using a dedicated GitHub repository at: https://github.com/coreywadsley/SeleST. Detailed and up-to-date instructions for installation and operation are included in the GitHub repository. An overview of key features and design choices is provided below.

#### Application

**Fig. 1:**
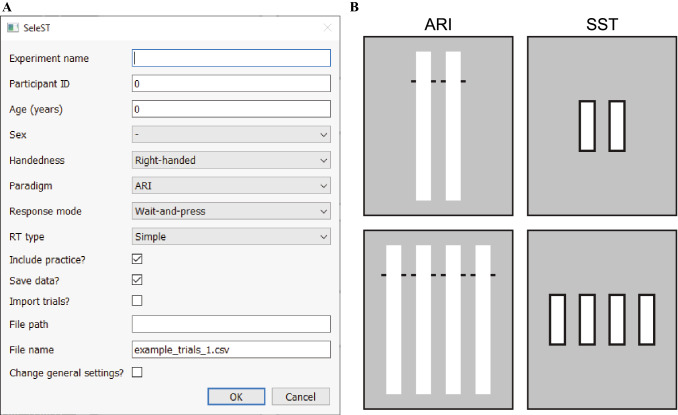
The selective stopping toolbox (SeleST). **A** A graphical user interface (GUI) is presented whenever SeleST is run. Participant demographics and basic design choices, such as paradigm type, can be set from the initial GUI. Advanced settings can be set through subsequent GUIs by selecting the ‘Change general settings?’ option. **B** Default implementations of simple *(top*) and choice (*bottom*) variants of the anticipatory response inhibition (ARI) and stop-signal task (SST) in SeleST

SeleST makes use of the object-oriented basis of Python. Classes and functions are contained in two primary components: *SeleST_initialize* and *SeleST_run*. Most parameters can be modified using a graphical user interface (GUI) presented whenever SeleST is run (Fig. 1A). The default menu presents users with dialogue boxes where general information (e.g., participant demographics) and options (e.g., paradigm) can be set. Additional options (e.g., trial numbers, response keys, stimuli colours) can be set as desired through advanced settings GUIs. The design of SeleST provides maximum flexibility without disrupting data collection for experimenters who are inexperienced with coding. Advanced users can also make use of the design by inserting modified versions of the various functions that handle specific aspects of the task (e.g., stop-signal presentation, feedback).

#### Paradigms

SeleST contains simple and choice variants of multicomponent SST and ARI paradigms (Fig. 1B). A go-signal is presented for only two visual indicators during the simple variants, whereas a go-signal is presented for two of four possible visual indicators during the choice variants. An element of choice is typically included in the SST to slow go-signal RTs and provide sufficient time for a stop-signal to be processed before the go response is enacted. Choice variants may also be beneficial to avoid the automaticity of responding that can occur in simple contexts (Verbruggen and Logan 2008). Simple variants may be more suitable for populations where response times are slowed (e.g., Parkinson’s disease) or for the ARI paradigm since it is robust to response slowing (Leunissen et al. 2017). Notably, the data output structure is consistent across all paradigm types, allowing flexible comparisons and design choices based on the population of interest.

#### Default task structure

The default version of SeleST implements a choice variant of the SST or ARI paradigm. Participants first complete a practice routine with in-built task instructions. During practice, a participant is first introduced and required to complete a block of only go-trials. Stop-trials are then introduced, and participants must complete a mixed block of go and stop-trials. Introducing go trials before stop trials works to prioritise “going” as the default mode of responding and ensure a measure of go performance that is not biased by the implicit expectation of stopping in mixed blocks (Verbruggen and Logan 2009b). A nonbiased measure of go response time is also important when determining if deficits in response inhibition are beyond simple differences in processing speed (e.g., Verhaeghen 2011). After practice, the default trial arrangement includes 288 go trials and 144 stop trials (equally distributed for stop-all, stop-left, and stop-right trials) across 12 blocks. Each block takes approximately 2 min to complete. Thus, a behavioural assessment of selective stopping can be completed within 30 min.

Custom task routines can be implemented easily within SeleST. For example, trial and block numbers can be modified using the GUIs to set a 25% stop-signal probability or to include stop-all trials only. More advanced customisation is made available through the option to import a custom trial list. A custom trial list can be created with the *SeleST_trialArrayCreator* script in which various parameters (e.g., cue and stop-signal colour) can be specified across a series of custom trial types. Custom trial lists are helpful for advanced implementations of SeleST, for example, to assess proactive selective stopping (e.g., Cirillo et al. 2018) or create a stimulus-selective stopping task (e.g., Sanchez-Carmona et al. 2021). Together, the above options provide a variety of methods for experimenters to modify the task structure to suit their demands.

#### Feedback

Trial-by-trial feedback is available for all paradigms in SeleST. Feedback is provided by points on a sliding scale, with more points earned by making more accurate responses. During the ARI paradigm, accuracy is determined by stop success or the closeness of the go-signal RT to the target time. During the SST, accuracy is determined by stop success or the speed of the go-signal RT. Points are awarded independently for the left and right responses to provide separate feedback for the respond-hand and stop-hand during partial-stop trials. The number of points awarded is signalled by changing the colour of the target line or stimulus outline for the ARI and SST paradigm, respectively. Using symbolic rather than text cues helps avoid potential language-related confounds. The current block and total score are updated and displayed to the participant at the end of each task block. An option to modify or disable trial-by-trial feedback is also included in SeleST.

Trial-by-trial feedback is beneficial to task performance. Strategic slowing (i.e., delaying responses to improve the chance of success in a stop trial) is a prevalent confound in response inhibition paradigms (Verbruggen et al. 2013; Verbruggen and Logan 2009b). The tendency to slow responses is less during the ARI than the SST since go-trial success depends on a temporally precise response (Leunissen et al. 2017). We have applied this concept to the SST in SeleST by making feedback during a go-trial dependent on the speed of the response, as opposed to the binary presence of a response used in typical implementations. We also use points for feedback rather than information on RTs or stop success. Points are a way to gamify a task which, in turn, can enhance task engagement and be more intuitive than information on response times for most populations (Lumsden et al. 2016).

### Experiment protocol

Choice variants of the ARI and SST implementations of SeleST were used to assess response-selective stopping. Participants were seated comfortably in front of an LG-24GL600F-B monitor (144 Hz refresh rate, ~ 60 cm viewing distance) controlled by a RTX A4000 Laptop GPU. The operating system was set to optimise timing as per the recommendations of Bridges et al. (2020). Responses were made using the middle and index fingers of both hands on a DELL KB216 keyboard. A keyboard was used for accessibility, but a specialised response box (e.g., Li et al. 2010) is recommended for accurate timing during advanced implementations of SeleST. Each trial consisted of a fixation (0.75–1.25 s), response (1.25 s), and feedback (1 s) period.

**Fig. 2:**
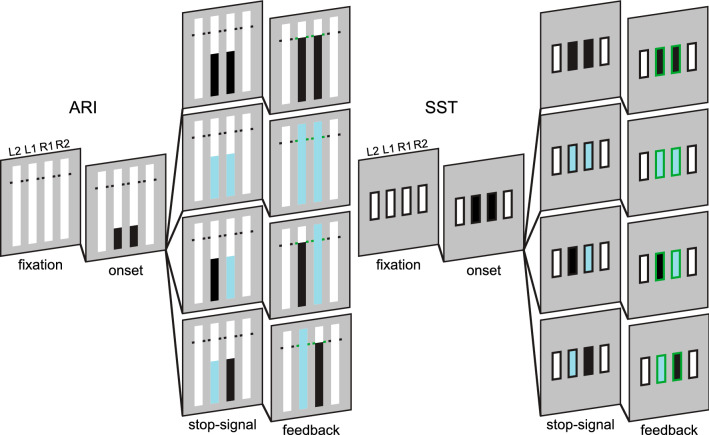
Schematic of primary trial types during choice variants of the anticipatory response inhibition (ARI) and stop-signal task (SST) in SeleST. Participants are presented with four empty indicators during fixation. A multicomponent ‘go’ response is cued for either the inner (L1 & R1, *depicted*) or outer (L2 & R2) indicators by presenting the go-signal (*ARI* filling bars reaching target; *SST* indicator turning black). Nonselective stopping is assessed by presenting a stop-signal (indicator turning cyan) on both the selected indicators. Selective stopping is assessed by presenting a stop-signal on either the left or the right indicator while the other continues as initially cued by the go-signal

Trial onset occurred when the go-signal was presented on two of four possible indicators. The go-signal was always presented for either the two inner or two outer indicators; thus, the choice component required responding with either the index (inner) or middle (outer) fingers. Inner and outer trials were randomised and distributed equally throughout the task. The objective during most trials was to respond with both hands (go-left go-right: GG). A subset of stop trials was included to assess response inhibition (Fig. 2). A stop-signal required the corresponding response to be withheld and was presented for both indicators during nonselective stop-all trials (stop-left stop-right: SS). During selective partial-stop trials, a stop-signal was presented for either the left (stop-left go-right: SG) or right (go-left stop-right: GS) indicator, requiring the response to be withheld in the stop-hand but not in the respond-hand. The stop-signal delay (SSD) was initially set to occur at 250 ms for SS, SG, and GS trials. The SSD was then adjusted in steps of 35 ms (~ 5 frames) across each stop trial type independently. The SSD was increased after successful stopping and decreased after unsuccessful stopping to obtain an average stopping success of ~ 50%.

Participants completed the SST and ARI (counterbalanced order) in a single experiment which lasted approximately one hour. Task instructions were built into SeleST and given prior to the start of each task. Participants completed a certain-go practice block (36 GG trials) and then a maybe-stop practice block (24 GG, 4 SS, 4 SG, 4 GS trials) for familiarisation. The task consisted of 12 maybe-stop blocks after practice, resulting in a total of 288 GG, 48 SS, 48 SG, and 48 GS trials for each paradigm. The trial order was randomised with the exception that each block started with at least one GG trial. Participants were informed that their primary goal was to earn as many points as possible and to avoid slowing their responses to prepare for a stop trial. The block and total score were updated and displayed at the end of each block. Paradigm-specific information is presented below.

#### ARI

The display for the ARI paradigm consisted of four white bars (15 cm high, 1.5 cm wide) on a grey background. A black horizontal target line was positioned behind each bar at 80% of its total height. Trial onset occurred when the two innermost or outermost bars appeared to “fill” (i.e., gradually turn black from bottom to top). The objective during GG trials was to cease the bars from filling as close as possible to the target lines. Each bar took 1 s to fill completely; thus, a target RT of 800 ms was cued during GG trials. Response keys were pressed with either the index fingers (inner bars) or middle fingers (outer bars) to cease filling. On stop trials, either both bars (SS), or only the left (SG) or right bar (GS), changed colour (cyan). Participants were instructed to let the corresponding bars fill completely when a stop-signal was presented. Points were awarded based on the closeness of the RT for each response (green [100 points]: < 25 ms or successful stop; yellow [50 points]: 26–50 ms: orange [25 points]: 51–75 ms; red [0 points]: > 75 ms or failed stop).

#### SST

The display for the SST paradigm consisted of four empty white bars with black outlines (5 cm high, 1.5 cm wide). Trial onset occurred when the inner or outer bars turned completely black. The objective during GG trials was to respond with the correct response keys as fast as possible. Either both, the left, or the right bar turned to the stop colour after the go-signal during SS, SG, and GS stop trials, respectively. Participants were instructed to cancel the corresponding go response when a stop-signal was presented. Points were awarded based on the speed of RT for each response (green [100 points]: < 400 ms or successful stop; yellow [50 points]: 401–500 ms; orange [25 points]: 501–600 ms; red [0 points]: > 600 ms or failed stop).

### Dependent measures

Data were processed using custom Python analysis scripts available in the SeleST GitHub repository. Responses during GG trials were coded as errors if the incorrect response keys were chosen or if part of the cued response was omitted. Mean GG response time was calculated across both hands during successful trials. Response slowing was calculated by subtracting mean GG response time during the certain-go block from the mixed go and stop task blocks to quantify strategic slowing. Response variation was calculated as the median absolute deviation of GG response times. Stop-trial data were taken from stop-all trials or averaged across GS and SG partial-stop trials for statistical analyses on nonselective and selective stopping, respectively. Mean stop success was calculated as the percentage of trials where the response was correctly withheld in the cued indicators. Mean SSD was calculated and made relative to the target for the ARI paradigm, thus, smaller and larger SSDs indicate less time available for stopping in the ARI and SST, respectively. Stopping-interference was calculated on a trial-by-trial basis by subtracting the mean GG response time during the task blocks from the response time of the respond-hand during partial-stop trials. Stopping-interference was used to determine stopping selectivity, where greater values indicate more interference (less selective stopping). The assumption of the independent race model (fail-stop RT < go RT) was tested for each paradigm across nonselective stop-all and selective partial-stop trials, respectively. Stop-signal reaction time (SSRT) was estimated with the integration method (Logan et al. 1984). Response times from GG trials with errors were included, and omissions were replaced with the maximum RT value (1.25 s) before averaging across both hands (Verbruggen et al. 2019).

### Statistical analyses

Data were analysed using JASP software (Version 0.16.3; JASP Team 2020). Evidence was determined using Bayes factor in favour of the alternative hypothesis (BF_10_), where values greater than 1 indicate support for the alternative hypothesis and values less than 1 support the null hypothesis. The strength of evidence was determined using a standard BF_10_ classification table (BF10 < 0.3: moderate evidence for null hypothesis; 0.3 ≤ BF10 ≤ 3: inconclusive evidence; BF10 > 3: moderate evidence for alternative hypothesis; van Doorn et al. 2021). Comparisons of GG trial (success, response slowing, response variation) and stop trial (success, stopping-interference) performance between the ARI and SST paradigm were made using Bayesian paired Wilcoxon signed-rank tests with default priors (van Doorn et al. 2020). The association between stop-signal delay and stopping-interference was determined using Bayesian Kendall’s tau correlations with pooled data from successful partial-stop trials across all participants. A frequentist linear mixed model with a fixed effect of Outcome (successful, failed) and random effect of participant was used to determine whether log-transformed stopping-interference was specific to successful partial-stop trials. In this case, stopping-interference data were also pooled from all participants. SSRT data were compared between nonselective stop-all and selective partial-stop trials with Wilcoxon signed-rank tests to determine how the speed of stopping is impacted by the required selectivity for each paradigm separately. Data are presented as median ± interquartile range unless otherwise specified.

## Results

### Trial success and response times

**Fig. 3:**
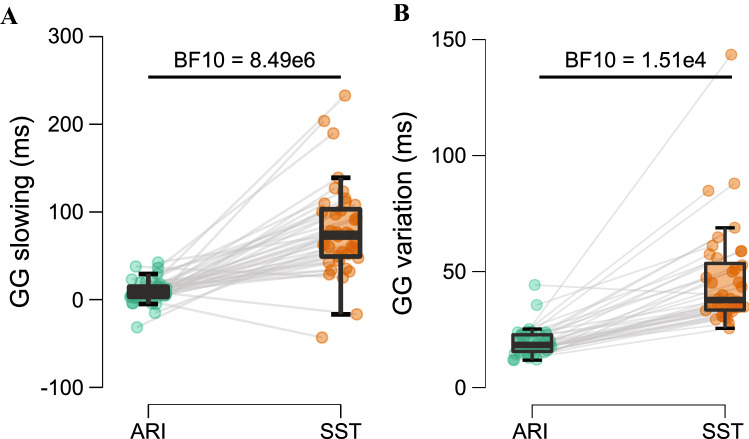
Go-left go-right (GG) trial behavioural comparisons between anticipatory response inhibition (ARI) and stop-signal task (SST) paradigms. **A** Response slowing measured as the difference in GG response times from the go-only to mixed task blocks, where values greater than 0 indicate more slowing. **B** Response variation measured as the median absolute deviation of GG response times during the mixed blocks. Grey lines connect paired participant averages

Trial success and response time results are shown in Table 1. There was no difference in GG success between paradigms for either the go-only (BF_10_ = 0.245) or mixed go and stop trial blocks (BF_10_ = 0.300). The amount of response slowing from the go-only to mixed task blocks was greater in the SST (73.6 ± 54.0 ms) compared to ARI (7.3 ± 11.6 ms; BF_10_ = 1.16 × 10^5^; Fig. 3A). The median absolute deviation during GG trials was greater in the SST (37.8 ms ± 20.1 ms) compared to ARI (18.4 ± 7.2 ms; BF_10_ = 1.94 × 10^5^; Fig. 3B). As expected, stop success was close to 50% for both paradigms, but overall was greater with ARI than SST for both stop-all (BF_10_ = 356.05) and partial-stop trials (BF_10_ = 5.38). GG trial success was similar, but responses were slower and more variable during the SST compared to ARI.

**Table 1:** Results for go and stop trials during ARI and SST paradigms

	GG (go-only)		GG (mixed)		Stop-all		Partial-stop	
	ARI	SST	ARI	SST	ARI	SST	ARI	SST
Trial success (%)	100.0 (2.8)	100.0 (2.8)	98.8 (1.5)	98.6 (1.8)	52.1 (2.1)	47.9 (4.2)	50.0 (1.0)	49.0 (4.2)
Response time (ms)	792.5 (8.9)	341.7 (45.5)	800.3 (7.7)	415.8 (61.5)	818.6 (21.7)	376.6 (50.2)	808.3 (12.2)	381.9 (55.4)
Stop-signal delay (ms)	–	–	–	–	256.6 (62.5)	166.0 (61.8)	239.5 (41.8)	172.5 (65.0)

Values reflect median (interquartile range). Trial success is calculated as the percentage of go trials where the correct response was chosen and the percentage of partial-stop trials where the response in the stop-hand was correctly withheld. Response time is calculated as the mean during successful go trials (Note: target time for the ARI paradigm was set to 800 ms). For stop trials, mean response time is calculated from trials where the response in the stop-hand was not withheld (i.e., failed stop trials). Stop-signal delay is calculated as the mean during partial-stop trials. For the ARI, values closer to 0 indicate less time for stopping, whereas values further from 0 indicate less time for stopping in the SST. *ARI* anticipatory response inhibition; *SST* stop-signal task; *GG* go-left go-right; *SS* stop-left stop-right; *GS* go-left stop-right; *SG* stop-left go-right

### Stopping-interference effect

**Fig. 4:**
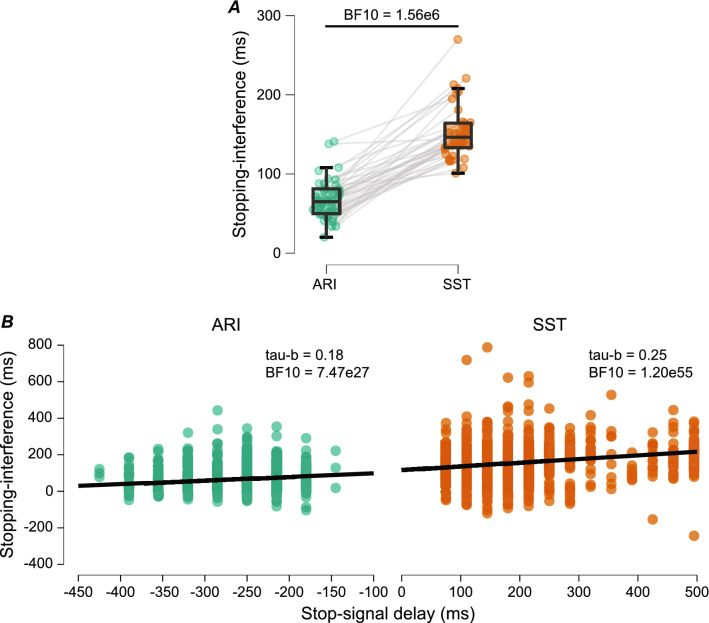
Comparisons between ARI and SST paradigms. **A** The stopping-interference effect calculated as the difference between mean GG response time and the response time of the respond-hand during partial-stop trials, where greater values indicate more interference. Grey lines connect paired participant averages. **B** Correlation between stopping-interference and stop-signal delay on a trial-by-trial basis. Stop-signal delay values closer or further from 0 indicate less time for stopping in the ARI and SST paradigms, respectively

The stopping-interference effect was examined from successful partial-stop trials. The stopping-interference effect was larger in the SST (146.5 ± 31.0 ms) compared to ARI (65.0 ± 146.5 ms; BF_10_ = 1.56 × 10^6^) and is shown in Fig. 4A. The magnitude of stopping-interference on a given partial-stop trial was positively associated with the stop-signal delay for both the ARI (tau-b = 0.18, BF_10_ = 7.47 × 10^27^) and SST (tau-b = 0.25, BF_10_ = 1.20 × 10^55^) paradigm (Fig. 4B). Stopping-interference was larger in the SST compared to the ARI paradigm and was larger when there was less time available for stopping.

**Fig. 5:**
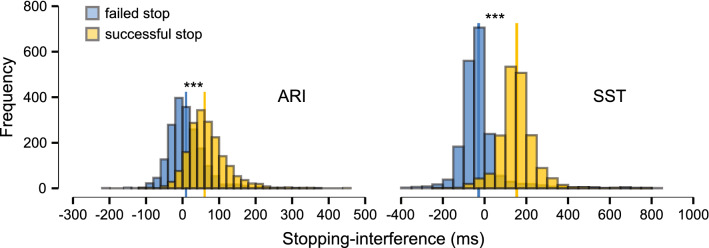
Frequency histograms (n bins = 25) of trial-wise stopping-interference from failed and successful partial-stop trials. Stopping-interference is calculated as the difference between mean go-trial response time and the response time of the respond-hand during partial-stop trials, where greater values indicate more interference. Solid vertical lines indicate condition means. ***P < 0.001 for fixed effect of Outcome (failed, successful)

Trial-wise analyses of stopping-interference (Fig. 5) indicate that response delays were greater during successful partial-stop (*M* = 62.3 ms, *SD* = 53.39 ms) compared to failed partial-stop trials in the ARI paradigm (*M* = 9.4 ms, *SD* = 48.8 ms; *F*_1,38.9_ = 165.1, *P* < 0.001). The same relationship of greater stopping-interference in successful partial-stop (*M* = 153.7 ms, *SD* = 82.7 ms) compared to failed partial-stop trials was also evident for the SST paradigm (*M* = -27.7 ms, *SD* = 107.9 ms; *F*_1,38.5_ = 210.8, *P* < 0.001). The stopping-interference effect was specific to successful partial-stop trials.

### Stop-signal reaction time

**Fig. 6:**
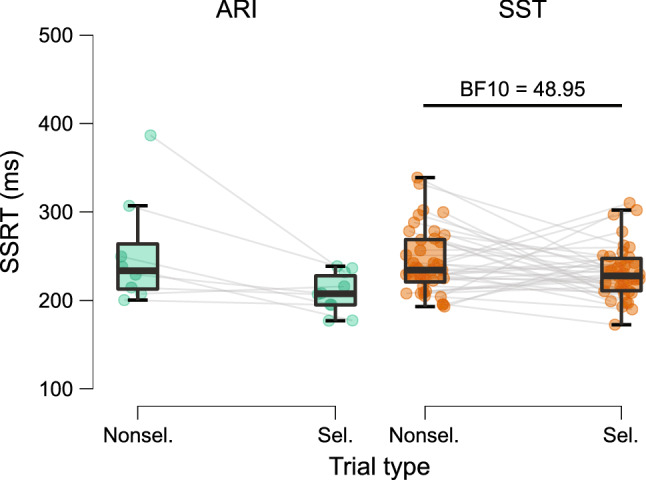
Stop-signal reaction time (SSRT) estimates during nonselective and selective stop trials for the anticipatory response inhibition (ARI) and stop-signal task (SST) separately. SSRT was only estimated for participants that met the independence assumption and was calculated using the integration method with inclusion and replacement of go trials errors and omissions, respectively. Grey lines connect paired participant averages. ***Posterior odds > 100

As expected, the independence assumption (fail-stop RT < go-RT) was met for 100% of participants during nonselective stop-all trials with SST. Conversely, the assumption was met for only 20% of the same participants with ARI. For selective partial-stop trials, the independence assumption was met in 95% and 25% of participants in the SST and ARI, respectively. After excluding participants who violated the independence assumptions, estimates of SSRT were larger in stop-all (243.8 ± 36.7 ms) compared to partial-stop trials for the SST (231.6 ± 30.6 ms; BF_10_ = 48.95). The ARI paradigm showed inconclusive evidence of SSRT differences between stop-all (254.1. ± 63.2 ms) and partial-stop trials (208.2 ± 22.7 ms; BF_10_ = 0.75), likely due to the limited number of available participants. Assumptions of the independent race model were met more frequently for the SST than for the ARI paradigm for both nonselective and selective stop trials (Fig. 6).

## Discussion

The present study compared response-selective stopping between multicomponent SST and ARI paradigms, using a novel open-source Selective Stopping Toolbox (SeleST). In support of the first hypothesis, go responses were more variable and slowed to a greater extent in the SST than ARI. In support of the second hypothesis, the stopping-interference effect was larger during successful partial-stop trials in the SST than ARI. The third hypothesis was also supported since the assumption of the independent race model was met more frequently in the SST than ARI. The findings indicate that nonselective and selective stopping can be assessed with SeleST using either the SST or ARI paradigm. Below we discuss the main findings and provide recommendations for future use.

### Responses are slower and more variable during the SST

Responses were more variable and slowed to a greater extent in the SST than in the ARI paradigm. While GG trial success was equivalent between both paradigms, response slowing from a go-only to a mixed go and stop trial context was ~ 66 ms larger in the SST. Strategic slowing in the SST has been attributed to the lack of a temporal constraint for GG trial success (Verbruggen and Logan 2009b). However, slowing still occurred in the current implementation of the SST, where points were awarded trial-by-trial based on response speed. Response slowing was small in the ARI paradigm, presumably since the go response was cued by a predictable indicator reaching a stationary target (MacDonald et al. 2017). This temporal constraint also likely contributed to response times being half as variable in the ARI than in the SST paradigm. Response slowing and variability are likely driven by the competing tendencies of going and stopping (Verbruggen and Logan 2009a). Strategic slowing does not invalidate the SST and instead likely reflects how individuals slow responses in contexts where stopping may be required (e.g., Hannah and Aron 2021). However, slowing does produce right-skewed response time distributions, which can bias SSRT estimates (Leunissen et al. 2017; Verbruggen et al. 2013). Isolating reactive and proactive response inhibition processes is also problematic with response slowing since successful stopping may simply reflect an omission of the go process rather than overt response inhibition. Therefore, the ARI paradigm provides for more stable GG trial performance.

### Stopping-interference is greater in the SST and specific to successful selective stopping

The stopping-interference effect was ~ 80 ms longer in the SST than in the ARI paradigm. Greater stopping-interference in the SST may be related to response slowing. Xu et al. (2015) identified that part of the measured response delay during partial-stop trials results from a sampling bias. According to the independent race model, successful partial-stop trials, from which stopping-interference is sampled, are likely to reflect the slowest components of the response time distributions (Logan et al. 1984). Thus, a portion of the measured response delay likely reflects a natural discrepancy between GG trial and successful partial-stop trial response times. While it is possible that a sampling bias also impacts stopping-interference estimates in the ARI paradigm, it is likely to be smaller since slowing is less than in the SST, and the assumptions of the independent race model are often violated (Leunissen et al. 2017). Therefore, the stopping-interference effect during partial-stop trials is present in both paradigms but may be inflated by a sampling bias in the SST.

The stopping-interference effect was specific to successful partial-stop trials for both the SST and ARI paradigms. A pause-then-cancel model of action stopping posits that a nonselective pause process is initiated during the presentation of infrequent stimuli, such as a stop-signal (Diesburg and Wessel 2021). Thus, a response delay would be expected to occur during all partial-stop trials, whether partial stopping was successful or not. However, the trial-by-trial analyses indicated that stopping-interference was specific to or at least amplified by successfully stopping part of a response. Greater stopping-interference during successful partial-stop trials is likely the consequence of a nonselective cancel process in addition to the indiscriminate pause process (e.g., Wadsley et al. 2022b). Stopping-interference was also positively associated with stop-signal delay on a trial-by-trial basis, whereby response delays were greater as there was less time available for stopping in both paradigms. Likely, excitatory processes in the respond-hand took longer to reach threshold the later into the planned response the stop process was triggered. Therefore, the stopping-interference effect is specific to successful selective stopping and temporally associated with stop-signal delay.

### SSRT was faster during selective than nonselective trials

In examining the reliability of SSRT estimates, the SST better conformed to the assumptions of the independent race model. The independence assumption predicts that response times during failed stop trials should be faster than go trials. This independence assumption was valid for all but two participants in the SST but only valid for ~ 25% of participants in the ARI paradigm. Violations of the independence assumption are problematic since they can produce unreliable estimates of SSRT (Verbruggen et al. 2019). The proportion of violations in the current ARI paradigm may have been elevated by adding a choice component, as previous studies have reported SSRT in many participants using a simple ARI paradigm (Leunissen et al. 2017; Zandbelt et al. 2013). It is important to note however, that the presence of a stopping-interference effect may violate the independence assumption since the presentation of a stop-signal should not influence the go process. Indeed, the independent race model is often violated in selective stopping contexts (Bissett & Logan 2014).

More sophisticated race models may need to be developed to better account for the relationship between going and stopping in a selective context (Bissett et al. 2021). Matzke et al. (2013a, b) have developed a Bayesian parametric model for SSRT estimation that can account for failures to trigger the stop process (Matzke et al. 2017) as well as context independence violations in a unimanual ARI paradigm (Matzke et al. 2021). An assessment of the efficacy of the above models during selective stopping is beyond the scope of the current study. However, SeleST provides a toolbox that facilitates such assessments across various contexts, which could be explored in future studies. An alternative approach to SSRT estimation is to use electrophysiological measures to capture stopping latency, for example, more direct measures from electromyography that indicate the time taken to cancel a response (Jana et al. 2020; Raud et al. 2022). In summary, the SST can be used more reliably than the ARI to describe behaviour in the context of the independent race model, but both paradigms may require alternative methods for selective stopping.

Analyses of SSRT estimates indicated that stopping was faster during partial-stop compared to stop-all trials in the SST paradigm. It is reasonable to expect SSRT to be longer during selective than nonselective stopping since it represents a more complex form of response inhibition that may occur through a slower but more selective mechanism (Aron 2011). Some studies have indicated longer selective than nonselective SSRTs (Cirillo et al. 2018; MacDonald et al. 2021). Other studies have found no difference, suggesting that a global response inhibition mechanism may be recruited regardless of stopping selectivity (Majid et al. 2012; Raud et al. 2020). In the current study, a trend for faster selective stopping was observed for the ARI paradigm, however, there were too few participants after exclusions. Faster SSRTs for selective stopping may result from participants attending more to partial-stop trials since they represent a greater percentage (22%) than stop-all trials (11%). A key distinction of partial-stop trials in SeleST is that feedback is provided for both the respond-hand and stop-hand, which encourages accurate selective stopping. Alternatively, the independent race model may not accurately reflect the relationship between going and stopping in a selective context, which requires more than global response inhibition (Bissett et al. 2021; Hall et al. 2022). In summary, the speed of selective versus nonselective stopping may depend on context. Future investigations may wish to investigate this matter by exploring alternative models of response inhibition (e.g., Matzke et al. 2013a, b).

### Recommendations for future use

The SST and ARI paradigms offer unique insights into selective stopping but may be suited to different experimental demands. Response slowing and variability were less in the ARI than in the SST, which corroborates previous comparisons made in a unimanual task context (Leunissen et al. 2017). Less response slowing is important when the stopping-interference effect is a key measure of interest since slowing can lead to estimates of stopping-interference inflated by a sampling bias (Xu et al. 2015). Tight control of response times in the ARI paradigm is also an important feature for studies investigating neural processes of response inhibition, for example, using transcranial magnetic stimulation to probe the primary motor cortex at specific time points during response preparation and inhibition (e.g., MacDonald et al. 2014). Indeed, stimulus timing can be set a priori relative to the target response time in the ARI paradigm, whereas it may be required to set them post hoc based on observed response times in the SST. Furthermore, acquiring a stable baseline can be more challenging when response times are variable based on pre-trial expectations. Thus, the ARI paradigm favours selective stopping experiments where strict control of response times is required.

The independence assumption of the independent race model was met in most participants for the SST but not the ARI paradigm. It is sensible to expect the SST to better conform to the independent race model since it was formulated from behavioural observations in a unimanual SST (Logan et al. 1984). Many race model variants have since been developed for reaction time paradigms that provide detailed descriptions of behaviour (Heathcote and Matzke 2022). For example, a parametric race model can be used to estimate SSRT as well as the percentage of stop trials where an inhibitory process was not triggered (Skippen et al. 2019). The applicability of the SST to race models is important for studies determining where deficits in inhibitory control may arise beyond simple behavioural measures (e.g., Choo et al. 2022). Overall, race models seem less applicable to selective stopping in ARI (Matzke et al. 2021). In summary, the assumptions of an independent race model are favoured by the SST when examining selective stopping.

## Conclusion

Selective stopping is a complex form of action stopping that can provide unique insights into response inhibition. Here we provide a freely available open-source selective stopping toolbox, *SeleST*, which can be used to assess response inhibition with either an SST or ARI paradigm. By directly comparing performance between SST and ARI paradigms, the current study showed that both paradigms might be best suited for different experimental demands. The SST is better suited when there is a desire to estimate SSRT in the context of the independent race model, whereas the ARI paradigm is better suited when strict control of response times is required. Importantly, we believe the SST and ARI paradigms can be viewed as complementary methods to assess selective stopping. The dual functionality of SeleST allows the decision of which paradigm to use to be guided by the research question. The research community interested in response inhibition can also drive advances with SeleST since it is freely available and open-source. SeleST can help bridge the gap between researchers who favour one paradigm over the other and may therefore increase the generalisability of selective stopping research.

## Data Availability

The datasets generated during the current study are not publicly available due to restricted ethical approval but are available on reasonable request. Analysis code is freely available along with an example dataset within the SeleST GitHub repository. The experiment was not preregistered.
